# Illuminating the brain: an interview with Karl Deisseroth

**DOI:** 10.1117/1.NPh.8.4.040401

**Published:** 2021-11-12

**Authors:** Yeka Aponte

**Affiliations:** a National Institute on Drug Abuse, National Institutes of Health, Baltimore, Maryland, United States; b Johns Hopkins School of Medicine, Solomon H. Snyder Department of Neuroscience, Baltimore, Maryland, United States

## Abstract

Yeka Aponte, a principal investigator at the NIH and an adjunct professor at Johns Hopkins Neuroscience, interviewed her mentor and colleague, Karl Deisseroth, research scientist and psychiatrist at Stanford School of Medicine, about his pioneering work in optogenetics and ongoing research.

**Figure:**
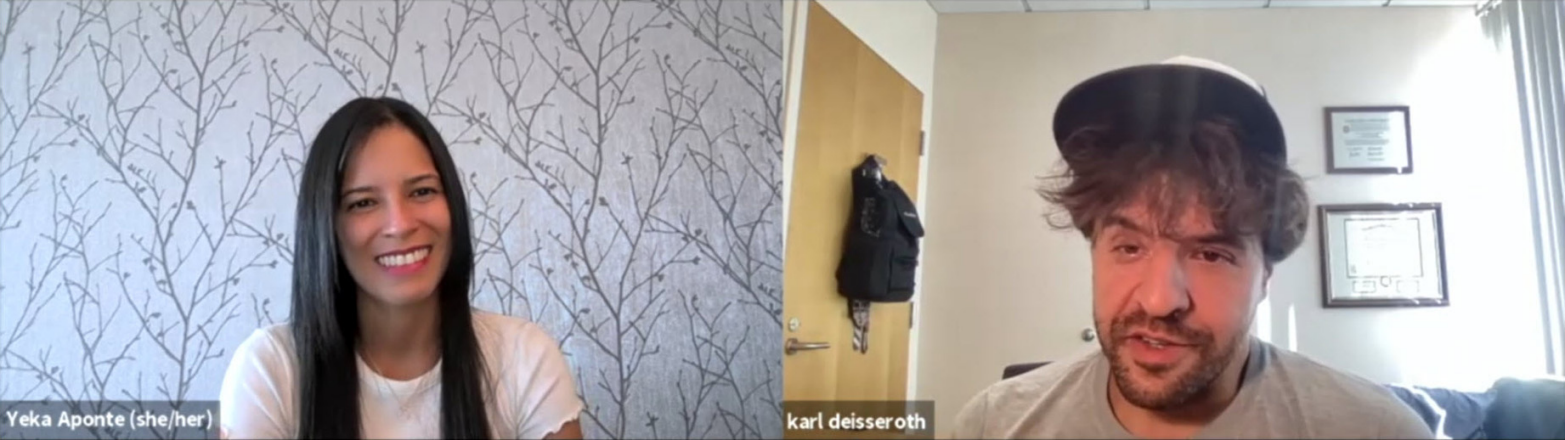
*Neurophotonics* associate editor Dr. Yeka Aponte interviewed Dr. Karl Deisseroth, professor of psychiatry and behavioral sciences and of bioengineering at Stanford University School of Medicine. Readers are also invited to view the interview video at https://doi.org/10.1117/1.NPh.8.4.040401

Yeka Aponte: Hello everyone, my name is Yeka Aponte. I’m a principal investigator at the NIH and also an adjunct assistant professor at Johns Hopkins Neuroscience. I’m very delighted to introduce Dr. Karl Deisseroth as our guest here at *Neurophotonics*. Dr. Deisseroth is a research scientist, a medical doctor, and a psychiatrist at Stanford School of Medicine. His laboratory has developed state-of-the-art tools to study the brain and how neural circuits drive behaviors essential for survival, but also to understand mental disorders. Welcome, Karl! Congratulations on your Lasker award! How do you do?

Karl Deisseroth: Thank you for the invitation and the introduction. I’m looking forward to talking. I’m excited by all the opportunities that the field is presenting to us and happy to talk about the future, past, and present. Really, the photonics and optics community has been central to everything we’ve done, so it’s a great chance to reach out and communicate—with the community!

Yeka Aponte: Indeed! So, let’s get started. You are a neuroscientist and a psychiatrist, but before that you were also a biochemist. What inspired you to study the brain?

Karl Deisseroth: Well, I think, even deeper, before any scientific training, I was interested in the brain. As a little kid, I was kind of introspective and thought about what was going on in my head, and then I really developed a love of writing and literature. I was curious about how words and sentences could make me feel, and I was interested in the communication between words and the brain and feelings, and probably that’s where I first wondered about the brain—could it be studied, could we understand feelings? But I didn’t have a clear path forward. Then, in college, I was exposed to modern genetics, modern biochemistry, and cellular level understanding of developmental biology, immunology, and I saw the opportunity of being able to work with cells, with molecular tools, with genetic tools, to understand complex systems that seemed intractable and just all the beautiful examples over the decades of other scientific fields making profound headway in seemingly hopelessly complex systems by a rigorous molecular and biochemical and genetic approach. That to me was very inspiring and I thought, well, maybe, maybe I can bring a molecular bent toward understanding things as mysterious as feelings.

Yeka Aponte: That brings us to my next question: why did you want to become a research scientist in academia, as opposed to working for a biotech or pharmaceutical company?

Karl Deisseroth: That’s a great question. I think I didn’t have in my life role models for biotech or industry opportunities. Obviously, it’s amazing what gets done in that realm—it’s long been true. It’s certainly true right up to the present, and hopefully into the future. I didn’t have that as an obvious path forward. I think I might have, though, gone down the medical route anyway. Because I was interested in research, I wanted to do an MD-PhD program and most people who do MD-PhD programs have academia in mind, at least initially. A lot of them end up making other decisions, but that’s kind of the typical progression. When I look at it now, one of the hats I wear is directing undergraduate education in bioengineering at Stanford, so it comes up all the time—students now are very curious.

Yeka Aponte: Yes, they have more options than we had, back in the day. They’re more exposed to different opportunities. Which is great, you know because we prepare them to be successful in whatever they decide to do next, not necessarily academia, right? They can still do science in biotech, but differently.

All right, so we both know that neuroscience is not a field where things can be solved quickly, which makes it very exciting. But what about psychiatry? What triggered your passion to become a psychiatrist? And not just that, but to combine both neuroscience and psychiatry into your research?

Karl Deisseroth: Psychiatry was also a case where I didn’t have any kind of role modeling. I had never considered it and it wasn’t even close to a possibility in my mind, all the way through medical school, until I got a personal exposure to it. I was interested in the brain, but I thought neurosurgery most likely. I thought about neurology, thought about anesthesiology even, thought about ophthalmology even, as a route into the brain. But I didn’t even consider psychiatry, not even for a moment. But it’s required that you do psychiatry as a rotation in medical school, and it just blew my mind. It was amazing. The need was so great, first of all—I mean the epidemiology of psychiatry—once I delved into it, I realized how much disability, how much suffering, and how much morbidity and mortality around the world is caused by psychiatric disease specifically—it’s not widely appreciated. And all over the world. And then also just the depth of the mystery. And then, finally, the questions that psychiatrists got to ask, even if they couldn’t answer them, the things they got to ask, the questions they were able to think about on a daily basis—they were the most interesting ones, and so everything kind of clicked for me in that moment of exposure to psychiatry.

Yeka Aponte: That’s interesting, because perhaps they couldn’t answer those questions because we didn’t have the tools. But the more we advance the more we’re able to answer those questions.

During this interview, we also want to show a little bit of your human side. We have heard that fishing is your favorite hobby.

Karl Deisseroth: Yes.

Yeka Aponte: If I remember correctly, it’s fly fishing? So how does such connection with nature inspire and shape your research interest—if indeed it does?

Karl Deisseroth: Yes, fly fishing is a great hobby for a biologist, because there’s a lot of biology in fly fishing. You have to think about the ecology and the motivational states of fish, their brain states, their signal processing, and their sensory detection systems. All that’s going on, but it’s also very physical, very active. Sometimes you’re fighting a very powerful current and you’re constantly casting and mending—also fine skills, too. It’s actually quite a physical challenge and a mental challenge. It’s not restful, but it can be peaceful, if that makes sense. So, it’s a way to connect with nature, in a very complex way that’s interesting.

Yeka Aponte: The prey, and the predator, escaping. You have to use the hypothalamus, right? So now, for those who are not familiar with your work, you’re very well known for developing state-of-the-art technology, such as optogenetics and CLARITY. Let’s start with optogenetics. Could you please explain in simple words how those light modulating opsins work?

Karl Deisseroth: Okay, this is really a gift that that nature gave us that we didn’t appreciate and for a long time, as is often the case. As it turns out, microbes have spent hundreds of millions of years evolving single proteins that do an amazing task: they turn a photon of light—they “transduce” it—into electrical current flow across the membrane of the cell. And they do this all-in-one with a single molecular machine. Of course, our eyes do something analogous, but they don’t do it with a single protein encoded by a single gene. We have a very complex signal transduction cascade in our eyes; first, the photon’s detected and then there’s signal amplification, and a number of steps until you get to the electricity. Microbes do it all with one single gene, single protein. These are called the microbial opsin genes, and they generate very tiny currents, and so that’s probably why they weren’t necessarily appreciated for the possible application that we were able to establish. What we found—and this took a number of years to develop fully to make sure it actually worked and was practical and versatile—what we developed is a way of using these natural genes that encode these single proteins, to give us the ability as experimentalists to turn on or off individual types of neurons, or even individual neurons in the brain of a living, behaving, complex animal, like a mammal. And that gives us this causality approach to neuroscience, with gain and loss of function—the same power as geneticists and developmental biologists and biochemists have had for gain and loss of function in their complex systems.

Yeka Aponte: With the ultimate goal to also try to apply these tools in, for example, psychiatry treatments?

Karl Deisseroth: I wouldn’t say that’s the ultimate goal, but that might happen. I see the real value of optogenetics as understanding. It’s a way to give us a deep, cellular-resolution, and causal understanding of the amazing things that happen in the brain. And that is the foundation you need for everything. It’s the foundation for basic science, but that’s how you could build any kind of therapy—once you know what matters, once you know what gives rise to symptoms, what ameliorates symptoms. It might be medication, it might be a noninvasive brain stimulation treatment, but at least now you’re grounded in this causal and precise understanding. That’s the real hope, just to build understanding of the brain. That said, people are looking at direct applications of optogenetics to the human brain. My friend and colleague Botond Roska in Switzerland succeeded this year in doing that. He successfully put a microbial opsin into a human retina of someone who was blind and was able to confer some light sensitivity, and the person can reach two items on a table that were invisible before. There was a restoration, effectively, of some level of vision in this blind person.

Yeka Aponte: That was amazing. And that links to this question, when you’re talking about structure: we know that another incredible technique developed your lab is CLARITY. Could you please tell us about the basics, and how such technique will potentially reveal microstructures in the human brain? I mean, we have so much information when it comes to the cytoarchitectural organization of the mouse and rat brain, yet very little is known about circuits in the human brain.

Karl Deisseroth: Yes, so this came along later. Optogenetics, we got up and running between 2004 and 2010 or so. Around that time though, it was very clear that we didn’t have the anatomical or wiring information that we would really like, to fully make sense of what we were doing in the brain. So, after some thinking, I developed a method that’s now broadly stated as hydrogel tissue chemistry. This is a way of using hydrogels, which are these soft hydrophilic water-like or jello-like polymers, and building these hydrogels within cells in intact tissue, everywhere within the cells throughout the tissue at once, and anchoring biomolecules of interest like RNAs or proteins to that gel, so you create a new composite structure: the hydrogel–tissue composite.

Then you can treat this new thing as a new kind of entity. You can transform it in various useful ways—for example, you can solubilize all the lipids, get rid of them. You could use strong detergents like SDS. Most of the opacity of the brain or any tissue, except for pigmented tissues, comes from light scattering at lipid–water interfaces, so by getting rid of lipids you greatly increase the transparency of the organ. So that lets you get light through, exchange light, and exchange information in an intact organ or intact tissue, while preserving this molecular and cellular level organization that’s so important. And not just light—you can send in enzymes. We found you can even do transcriptomic-level sequencing of individual cells within intact tissue in this hydrogel tissue chemistry framework. After our initial version of this, which we called CLARITY in 2013, there’s been a big profusion of methods. Not just from our lab—we’ve done a lot, including this latest method called STARmap—but many other labs around the world have built on this and have their own variants, so there’s been an exciting growth stemming from the initial building of these hydrogels within the cells of intact tissue.

Yeka Aponte: As you said before, it’s a systematic structure from molecular, cellular circuits, to behavior—and then how this behavior gets disrupted, for example. Now, basically, the use of photonics and photonic engineering has been key for performing these optogenetic experiments and for using CLARITY. Could you tell how this has helped your research?

Karl Deisseroth: Yes, it’s been essential. I briefly alluded to the realization that these microbial opsins had been known for decades, but there was a convergence of technologies that we were able to bring together at the right moment, and a lot of these came from other fields that I had nothing to do with at all. And one of them, for sure, is photonics. For example, LEDs were not as strong and didn’t generate as high irradiances as they do now, and the laser diodes that we were able to make optogenetics in living mammals operational with were actually a crucial step in the whole thing. We didn’t have even a way to get the right light in at the right power without the laser diodes that we’ve developed coupled to fiber optics, to create that neural interface. We described that interface in 2007 and that’s still the workhorse of optogenetics, around the world, this fiber optic interface.

At the same time, we and others had gotten really interested in single-cell-resolution optogenetics, and that brought in a whole other field of optics and photonics. If you want to get to single cell resolution in the mammalian brain, you’ve got to use two-photon microscopy most likely, and so that was a whole advance that was critical to get to the single-cell level, which we described in 2012 in the living mouse brain. And then the question was, could you start to control not just one single cell, but many single cells, ensembles, all at once, dozens, or hundreds? A number of approaches have been developed for that, coming from the photonics community. One way that we’ve worked on hard is using spatial light modulators to generate 3D holograms of spots of light in tissue and we can use that to control even hundreds of individually specified cells in living animals, and control specific perceptions, specific actions, and understand the causal relationships of these cells to see how the brain works. Those are just a few examples—there are many others. Most of what we do would not have been possible without the photonics community.

Yeka Aponte: I do agree, and for crying out loud, I remember writing a review article recently about functional imaging and the advancements, and I heard rumors that a two-photon system for freely moving mice is on the make, and as a matter of fact, it’s already out! So that is very impressive. We were limited to still image with two-photon in head-fixed mice, and now we’re able to do it in freely moving mice. To me, that is just amazing—in such a short period of time!

Besides very fundamental questions, what other pioneering research in either neuroscience or photonics set the stage for you to develop optogenetics?

Karl Deisseroth: This convergence of technologies from different directions was critical. I mentioned the photonics side, but I’ll talk about some other branches as well. One was the whole microbial opsin community. I’ve become embedded in that community. I have a lot of very dear friends and colleagues in that community now, like my friend Peter Hegemann, who was a co-laureate with the Lasker this year. He’s a fantastic guy and we’ve had many wonderful collaborations. He’s been working on these proteins for many years and getting to know him, getting to know that community, and becoming part of it has been really a thrill—particularly because it’s enabled me to do biochemical-level work, which is where I started, as you mentioned earlier. So, we’ve been getting the high-resolution crystal structures of the major classes of channelrhodopsins, for example, we’ve been doing that for the last 10 years or so, and cryo-EM structures, most recently. Making modifications, point mutations, carrying out structural modeling of molecular dynamics—all of that builds fundamentally on this wonderful community of microbial biologists, biophysicists, membrane biophysics researchers, and people studying the detailed photocycle of these proteins, and that has been just a fantastic part of what we’ve been able to do.

One other field I’ll mentioned is virology. That was also critical. Because there are such tiny currents that are generated by microbial opsin gene-encoded proteins, by comparison with more “professional” mammalian voltage-gated sodium channels, it was very clear that with a conventional transgenic approach, where you have five to seven copy numbers typically, we weren’t going to get strong, robust actuation of the cells of interest. But in the 90s, and particularly late 90s, there had been a lot of development of viruses, lentiviruses, adeno-associated viral vectors, making them safe, making them modifiable in tractable ways. I had worked with some of these viral methods right at the end of my postdoc, and becoming conversant in those was absolutely critical, because then I was able to bring these viral tools together with the microbial opsin genes, and generate this very versatile, high-copy-number, high-potency building block that the community could use. I’m very grateful to all these different communities that allowed us to bring all the threads together.

Yeka Aponte: Yes, everything you said highlights beautifully the importance of a multidisciplinary approach, working together in collaboration. Especially now during this pandemic, we realize that international collaborations are so important. To huddle! Now, do you mind sharing some of your deepest questions for both psychiatry and neuroscience?

Karl Deisseroth: My deepest questions? Well, that’s a deep question. As a psychiatrist, I treat patients with depression, severe and treatment-resistant depression. These are people who come to me who have tried in the outpatient community, have been through four or five or more courses of medications, or even electroconvulsive therapy, and things are not working. These are patients at the end of their options and suffering terribly. The other kind of patient I specialize in is autism, adult autism-spectrum-disorder patients. And these are also, like the depression patients that I work with, very hard to treat. There’s no medication that corrects the fundamental symptoms of autism. I can help them, but I can help them by mostly treating their associated or comorbid symptoms. A lot of them are very anxious, because the world is a very anxiety-provoking place for them. There’s a lot that’s inexplicable or confusing about human interaction and the natural flow of things in the world, and so these are very difficult patients. I help them, not enough, but a little bit. When I’m sitting there and I’m talking to them and they’re expressing their symptoms—like with autism, this feeling of being overwhelmed by the information rate coming in, by all the things going on in the world, not just the social signals, but sounds and other information from the world—I think there’s a very deep question there: how is it possible that we can do the things that we do? How do we handle a social interaction? How do we make sense of the incredibly complex content of speech and eye contact and group dynamics? It’s almost impossible if you think about it. One thing I’d like to understand is how we handle and use these very high information rates. Are we pre-modeling things in some way? And there’s a lot of evidence that in fact is going on. Our cortex is working very hard to predict what’s going to happen, rather than just react to it. I’d like to understand in a very deep way what that predictive role of the cortex is, how it’s operating, how that goes wrong in autism, where the prediction and the information overload seems to be such a fundamental part of it. I think that’s a deep question. As humans, we’re pushing the limits of what neural circuits can do, and maybe that’s why we get tired after too much social interaction.

Yeka Aponte: I agree. I think that we could say at this point that you’re learning from your patients how the brain works. If so, if that statement is accurate, what have you learned thus far from your patients, when it comes to the brain and how the brain works?

Karl Deisseroth: Patients… you know, as I’ve helped them a little bit, I think they’ve helped me more. They’ve helped me focus my attention on what really matters. They’ve helped underscore what the most critical things are and what, for the human brain, some of the most interesting things are, like this information issue. My patients with depression have taught me how disturbingly—cleanly—positive feeling can be removed from life. This is a very perplexing, shocking thing, that you can have a human being, who is not just sad, not just upset, but—just like your sense of smell or taste goes away when you have a cold—for these people who are suffering from depression, the ability to feel positive about things is gone. It has been deleted from their life. That’s one thing, and then there’s the imposition of negative feeling, psychic pain, on top of that, and so you’ve got these two things taking away pleasure and adding this internal suffering.

What I’ve just described, you don’t necessarily see that if you open the manuals and you read the symptoms of depression. You’ll read a list of things. You’ll see that they have to have five of these nine symptoms, to meet the criteria for depression. But the patients really highlight for me what really matters, what’s really causing the suffering and the disability, and that’s helped us focus our laboratory research on these questions. How does valence get attached or detached from experience, positive or negative? It’s good for the students and postdocs in the lab that I can reflect those conversations and those insights in ways that are helpful.

Yeka Aponte: Do you envision the use of optogenetics for the treatment of psychiatric disorders? When I think about the tools that psychiatrists have nowadays with their patients—for example, when I think about anorexia—we know that psychiatrists rely on words or “talk therapy” (I don’t know if that’s the right term to use) as a primary treatment, but what else? Is there anything we could use rather than, or together with, talk therapy?

Karl Deisseroth: The eating disorders are also, right now, not really treatable with medication. Again, we use medications, but it’s often to treat the comorbid symptoms. Many patients with eating disorders are depressed, and so they’ll receive antidepressants. There’s some indication that other classes of treatments, like the atypical antipsychotics, may facilitate the remapping of cognition that happens with talk therapy. In patients with eating disorders, there’s this initial tangle of social drives competing with primary survival drives for nutrition. Later it becomes more complicated. The behavioral pattern becomes really embedded and becomes even less about a social drive anymore. It almost takes on a life of its own and seems to become embedded with habit-forming circuitry and these very powerful rhythmic and counting activities—you know in neuroscience we almost see these as striatum-related activities, rather than sort of high-level cortical social interactions, and these are very hard to treat. Medications don’t really get to the core of the eating disorder, but over time you can start to shift these cognitions with talk and behavioral therapies, and the medications can help a little bit along the way. But to your question: will the deeper understanding that comes from optogenetics about what is truly causal, will that end up helping patients, like the eating disorder patients? I think it very well could.

Yeka Aponte: Yes.

Karl Deisseroth: We’re doing experiments right now in the lab—and we’ve published some of these—where we have actually found cells in locations like in the orbitofrontal cortex, where social cells inhibit feeding cells. We can identify exactly those cells. We know they’re causal and we know they’re naturally active, and if we get to know those cells better, we might identify even better medications based on what receptors they express, and so on.

Yeka Aponte: And we can deal also with the side effects. Most of these medications, they do work, but the problem is the potential side effects that they have. That’s why also people get scared—they’d rather deal with the disease than the side effects from the medication.

We also know that public awareness about stigma when it comes to mental illness has been growing exponentially throughout the last decade. In my opinion, stigma is one of the major challenges we face, not only in psychiatry but also for the treatment of these mental illnesses. How do we communicate to the general population that, for example, addiction, anorexia, or depression are not a choice, but they are a brain disease?

Karl Deisseroth: I think about this all the time. I’m glad you asked that. I didn’t know you were going to ask that, but it’s something I feel strongly about. As psychiatrists and as neuroscientists, we have to communicate with the public about this. What’s happened in the biology has been so exciting in bringing us to this material, physical understanding of where these symptoms can come from, that we have to communicate with the public about this. One thing I’ve done, just this year a book came out that I wrote called *Projections*.

Yeka Aponte: I have a question about it…

Karl Deisseroth: We will wait until later, but basically that’s my effort to reach out to the public, to try to communicate to everybody, not even people primarily interested in science, but everybody, to let them know what’s been happening. That, I think, will reduce stigma, if people know and can appreciate how far we’ve come in the last few years, and how that illuminates psychiatry and mental illness. I think that’ll be good for the whole community.

Yeka Aponte: You use the proper words, you know, because at some point, we have to stop using that jargon that we’re so used to as scientists, so that we can relate to the general population. Speaking of communication and, of course, your book *Projections*, how do you use social media to communicate your science to the public, if you use social media?

Karl Deisseroth: I do only a little bit of it. I have a Twitter—I’ve had it for I think six years and I’ve only tweeted about 300 times, which is pretty low rate, so I need to get better at it. I do think it’s hard to communicate complex concepts well in the social media platforms that I’ve seen, and so I haven’t tried to use them to engage in detailed conversations, because those seem to go wrong very often, and I don’t want to create misunderstanding or confusion. I mostly use it just to draw people’s attention to where they can find more deep treatments of things. Now, maybe I need to be better at that, I don’t know. What do you recommend? What’s your perspective on this?

Yeka Aponte: I asked you that question purposely to get advice from you, because I’m guilty as charged: I’m no good at social media. I mean, I use Twitter less than you do. Of course, I get info about the latest papers and things like that, but I certainly think that social media is changing the way that science is communicated. You know, as you said, I have yet to become really good at it. I’m always afraid that some of my comments could be misinterpreted. And we have seen it, not only with science, but we can see it with politicians and celebrities. You know, one has to be very careful about doing this, but I think it has helped the trainees a lot, when it comes to job markets and things like that. I keep going back to how can we use social media to remove stigma, to use words that will be understood by the general population, to make them understand that mental illness is a brain disease, not a choice. And then maybe we will get also more support from governments and politicians, funding to study these diseases.

Karl Deisseroth: Yes, I think that could help. The trick then is, how does a scientist develop that broad following? I think most of my followers are scientists, and so for your correct and lofty goal here, it’s not quite the right thing because I don’t necessarily have a direct route to the general public. So other routes are important to build that, like the lay public books and public talks. Whenever I get an invitation for a talk to the public, I try to accept it because I think that can have a big impact.

Yeka Aponte: That is great. Now again, your human side. I need you to tell me, how do you do it? You run a large group in your lab, you have a lot of collaborators nationwide and overseas. You see and treat patients. You’re a family man, you’re married, and you have five children—five! What is your strategy for keeping a healthy work–life balance? Do you mind sharing your advice with scientists that have children?

Karl Deisseroth: I wish there was anything simple to say. You know each day is its own puzzle to be solved. First of all, I’m just so fortunate to have people around me that help make it work. My wife’s a very accomplished physician-scientist herself. We’re in an environment where our kids’ schools are close, and we’re able to make things work that way. Each day, I get up early in the morning, I make breakfast, I make the lunches, and then do the drop-offs of the kids. And then later in the afternoon, there’s a complex well-orchestrated dinner choreography that happens and we all try to pitch in and help, but every day there’s some new challenge that comes up. We’re both doing clinical, trial-related work now and emergencies come up, where you’ve got just got to do something and the other partner, if they’re not doing something absolutely critical at that moment, they have to drop everything and take care of things. So, it is important to have that sort of relationship if it’s possible. That’s not possible for everybody, and I completely understand that. I will say, though, I’m grateful for what I have.

I do have some thoughts, though, that do help. One is that it helps if the different parts of your life at least can seem as though they’re working together. And this is something that a lot of MD-PhDs run into trouble with: they have a clinical practice, and they have their lab research, but they’re kind of different—they’re not pushing along the same axis exactly, and then that becomes a stressor. You’re being pulled in different directions. You go to do one thing, and you say “the more I work on this, the better I’ll be in this,” and you do the other thing—“oh, the more I work on this, the better I’ll be in that”—and then, it creates this conflict and it makes you unhappy and you end up abandoning one of them. So, what I try to do is, for all these different domains you mentioned, you know, the teaching and the writing and the science and the medicine, at least there’s a cover story that they’re all sort of helping each other, they’re all kind of pushing in the same direction. That helps internally, psychologically, and practically, too. So, I would just give that advice: if it’s possible—if your life can tolerate that level of complexity, which not everybody’s, for very good reasons, can—try to make sure that the parts are kind of pushing together at least a little bit—and that helps.

Yeka Aponte: Yes, it is very important to have very open and clear communication with your partner, so that you both understand the schedule, and then to compromise, a little bit, right? In the morning, as you said, you prepare brekkie, get the kids to school, and then in the afternoon, when you’re in the office or seeing patients, she can pick them up. I like to call both you and Michelle the “power couple of science,” because you are indeed a power couple, you know? I mean, you make it look easy from the outside, but I know that people have their dynamic and somehow you make it work. I appreciate very much that you’re sharing this advice, especially with young investigators. As you know, I don’t have children, but I see it, with my friends and the couples that have kids, and now during this pandemic, I can see the challenge of multitasking at home, but then you never switch off because you’re constantly at home on your computer. Then you’re like, “okay, I’m at home, but it’s one o’clock in the morning and I’m still replying to emails.”

During our last minute or so, I would like to highlight your book *Projections*. I just got it two or three weeks ago, and you know I’m a very slow reader—don’t ask me how your book has kept me hooked, because I usually say to my friends when they recommend me a book, “I’m going to wait for the movie to come out.”

Karl Deisseroth: You’ll have to wait a long time for this one!

Yeka Aponte: I think it’s a masterpiece. It’s beautifully written, and of course, I want to compliment you, because you are an amazing writer. Do you like writing? Did you learn how to write before becoming a principal investigator? Because I feel that, when it comes to myself, my writing has become better now that I’m a principal investigator. So, did you have that passion for writing before?

Karl Deisseroth: Yes, I love writing. It’s always been a passion of mine, from very early on. I’ve just loved forming words, finding the right word. I’ve loved the rhythm of sentences, and the rhythm of paragraphs. I’ve loved playing with sounds and sequencing.

Yeka Aponte: Poetry, right?

Karl Deisseroth: Yes, absolutely. But of course, it wasn’t my career. Actually, I did entertain that early on, before I got more interested in other things, but I’ve loved it all the way through. I love scientific writing. With writing *Projections* and letting myself be more creative in the use of words, I discovered how much I really love the artistry that you have the opportunity to do when you’re freed from typical scientific writing. And it became addictive! I was so much looking forward—I tried to block out two hours a day for a couple years, which is when I got the writing done. But I would look forward to it so much. Even if it was late at night or early in the morning, I’d be just desperate to get to the laptop and start working on the next paragraph, the next sentence, or even just find the right single word. It was amazing. So hopefully I’ll be able to do it again.

Yeka Aponte: I do love the cover, by the way—very colorful. It’s beautiful. *Projections* is a great read. What was the hardest part of writing your book? What were the key elements that you added to connect with the general audience, yet keeping the hardcore science?

Karl Deisseroth: Each chapter is centered on a human story, a human being who’s entered into an altered state. I think that was the critical thing: every human being loves human stories. They want to hear about other people, their experiences. That’s how we remember things, that’s how we care about things. And so each chapter is basically a story about one or two people. It starts that way but then the altered state that that person is in—whether it’s a grief or mania or an eating disorder—these become branching off points. Their symptoms, their experiences, their feelings—they become the branching off points into the science, into human history even, and prehistory, evolution, and also some of my own experiences, too. But it’s all centered on these human stories, and that I think is an important way to communicate with the public. By thinking about the patients and their experiences, of which there are many diverse and complex stories, and then the opportunities from science like optogenetics, and the things relating to it and what they have provided, I was able to build for each chapter a sort of unified structure that made a coherent point. So that was that strategy: taking human stories and linking to the science. There might be other ways of doing it, but that was what I settled on.

Yeka Aponte: It’s good. I don’t want to give away too much about your book (spoiler alert!)—I’m sure that you wrote some of these things in there—but what are you predicting for this research in the near future? In other words, what is cooking in your lab right now?

Karl Deisseroth: Well, we’re doing a lot more photonics-related development. We’re using these spatial light modulators to activate many hundreds of individual cells. We’re increasing our field of view over which we’re operating. In mammals, it’s over millimeters. We’re even reaching whole-brain, single-cell-resolution level in zebrafish, where we can now treat the entire brain as accessible at single-cell-resolution, to play-in and read-out information across the brain during behavior. Getting to this wider, more global perspective, while maintaining cellular resolution, is where we’re going now. That’s something that seemed very hard 10 years ago. It’s amazing that it’s accessible now and it’s really exciting. Of course, that brings all kinds of interesting other challenges—computational and data challenges—so we’re working on all those as well.

Yeka Aponte: And the deeper structures in the brain, right? You mentioned zebrafish—it’s beautifully clear and laminated, thus imaging should not be so complicated—but when you think about amygdala and hypothalamus, the challenges are there. Is this something that you are now developing in your lab, together with photonics engineering, to get to that level of resolution?

Karl Deisseroth: That’s right, we are working on getting to deeper structures. One thing that’s helped is the microbial opsin structures and genomics that we’ve been doing. We’ve identified some very light sensitive opsins that give us much better access to deep structures, less invasively, more cells. And we just got the high-resolution structure of one of them, called ChRmine. This is one of the pump-like channelrhodopsins. Now that we’ve now got its structure, we’re able to enhance it for new kinds of functions, with structure-guided engineering. So, all these different threads kind of come together in this moment as well.

Yeka Aponte: Exciting times! One last question: could you please share your words of wisdom with early career investigators, in particular, female scientists, as well as underrepresented minorities in STEM?

Karl Deisseroth: I really appreciate the challenges that are faced these days, and the pandemic hasn’t helped at all. The complexities of family structure have been stressed by the pandemic and by everything else going on in the world. Employment, the funding challenges, the conflicts that arise between all these different existential needs that people have are very severe right now. First of all, I very much empathize about the challenges that are being faced. And—some of this comes out in the book, too—for a while I was a single dad experiencing a lot of these very severe stressors, very personally difficult times in my early career, where existential situations were being experienced. “Can I continue? Maybe this is not a career I can follow through on…” So, I think the critical thing within the realm of science is to make sure that that you’re heading toward something that really is your passion. Where’s something about the problem or the field that you’re heading toward, where you still have time to do a course correction if needed, that you really, truly love, that you see as beautiful, and that excites you? You want to think about it, you want to wake up thinking about it, you want to go to sleep thinking about it. That will help so much. I ’ve seen many of those forks in the road, especially in the early career situation, where it might be an easy path that looks like it brings you to a reward that’ll help things progress a little further, more immediately, more quickly—just look at those carefully. Make sure that you truly care about that direction, make sure that you love it. Maybe, if it’s not perfect—because nothing ever is—try to see how you can morph it so it’s something that you truly love. I think that helps unify the conflicts into a more coherent whole. It’s not always possible, but for me that was critical, to try to think about how to structure things so that things were pushing in a way that fit with my passion.

Yeka Aponte: I really appreciate that you shared this because, from the outside, everybody sees you as this incredibly successful person. But, on the other hand, I want people to know that you’re very humble and you’re very supportive. As an underrepresented minority, I have been very supported by you. When we reach out, you’re always there, even if you’re busy, you let us know. And I want people to know this. When I think about my upbringing and my career—how can I be what I cannot see? I mean, when I think about being first of all, Latino descent, or Latinx, as they call it now, and also a female—we don’t have so many role models, you know? But also, I have been lucky that people like you—my “very successful white males,” let’s put it that way—have been using their voices for things that they value and for those like us that sometimes feel like we have no voice. I think that is just really remarkable. I’m glad that you’re sharing all that.

Karl Deisseroth: Well, thank you for saying that. I hope to do more, but it’s nice to know that there’s some positive impact. I’ve been so enthralled by the diversity of science, of neuroscience, of photonics, and how it’s increasing, how new ideas, new experiences, new ways of looking at things are coming from all over, and I hope that continues. I’ll do my very best to make sure it continues to.

